# Chemically Defined and Xeno-Free Cryopreservation of Human Adipose-Derived Stem Cells

**DOI:** 10.1371/journal.pone.0152161

**Published:** 2016-03-24

**Authors:** Melany López, Roni J. Bollag, Jack C. Yu, Carlos M. Isales, Ali Eroglu

**Affiliations:** 1 Department of Neuroscience and Regenerative Medicine, Medical College of Georgia, Augusta University, Augusta, Georgia, United States of America; 2 Department of Pathology, Medical College of Georgia, Augusta University, Augusta, Georgia, United States of America; 3 Department of Surgery, Medical College of Georgia, Augusta University, Augusta, Georgia, United States of America; 4 Orthopaedic Surgery, Medical College of Georgia, Augusta University, Augusta, Georgia, United States of America; 5 Department of Obstetrics and Gynecology, Medical College of Georgia, Augusta University, Augusta, Georgia, United States of America; The Ohio State University, UNITED STATES

## Abstract

The stromal compartment of adipose tissue harbors multipotent cells known as adipose-derived stem cells (ASCs). These cells can differentiate into various lineages including osteogenic, chrondrogenic, adipogenic, and neurogenic; this cellular fraction may be easily obtained in large quantities through a clinically safe liposuction procedure. Therefore, ASCs offer exceptional opportunities for tissue engineering and regenerative medicine. However, current practices involving ASCs typically use fetal bovine serum (FBS)-based cryopreservation solutions that are associated with risks of immunological reactions and of transmitting infectious diseases and prions. To realize clinical applications of ASCs, serum- and xeno-free defined cryopreservation methods are needed. To this end, an animal product-free chemically defined cryopreservation medium was formulated by adding two antioxidants (reduced glutathione and ascorbic acid 2-phosphate), two polymers (PVA and ficoll), two permeating cryoprotectants (ethylene glycol and dimethylsulfoxide), a disaccharide (trehalose), and a calcium chelator (EGTA) to HEPES-buffered DMEM/F12. To limit the number of experimental groups, the concentration of trehalose, both polymers, and EGTA was fixed while the presence of the permeating CPAs and antioxidants was varied. ASCs suspended either in different versions of the defined medium or in the conventional undefined cryopreservation medium (10% dimethylsulfoxide+10% DMEM/F12+80% serum) were cooled to -70°C at 1°C/min before being plunged into liquid nitrogen. Samples were thawed either in air or in a water bath at 37°C. The presence of antioxidants along with 3.5% concentration of each penetrating cryoprotectant improved the freezing outcome to the level of the undefined cryopreservation medium, but the plating efficiency was still lower than that of unfrozen controls. Subsequently, increasing the concentration of both permeating cryoprotectants to 5% further improved the plating efficiency to the level of unfrozen controls. Moreover, ASCs cryopreserved in this defined medium retained their multipotency and chromosomal normality. These results are of significance for tissue engineering and clinical applications of stem cells.

## Introduction

Stem cells with the capability of self-renewal and directed differentiation into mature cell types offer unique opportunities for regenerative medicine. However, there are differences between stem cells. In general, isolation and *in vitro* expansion of most adult stem cells pose logistic challenges, making the therapeutic use of stem cells difficult. Successful clinical applications of stem cells rely on their ability to be (1) isolated easily and in large numbers from a suitable source, (2) expanded *in vitro*, (3) non- or hypoimmunogenic, (4) multipotent, as well as (5) the ability to integrate into and interact with the host tissue. The so-called adipose-derived stem cells (ASCs) that are found within the stromal compartment of adipose tissue fulfill most of these criteria[1]. ASCs can easily be obtained in large quantities through a minimally invasive liposuction procedure that is routinely performed in clinics worldwide with over 350,000 annual procedures in the U.S. alone[2]. Hence, the autologous use of ASCs is feasible. Their allogeneic use is also possible because ASCs are not immunogenic and exert immunosuppressive effects as demonstrated by their action on controlling graft-versus-host disease[3,4,5,6]. In addition, ASCs possess considerable proliferative capacity and can be expanded for longer periods of time than bone marrow-derived stem cells[1,7,8]. The plasticity of ASCs has been demonstrated by inducing their differentiation into various lineages including osteogenic, chondrogenic, adipogenic, and neurogenic[1,9,10,11,12]. Moreover, the beneficial effect of ASCs on the healing of different diseases including acute myocardial infarction, peripheral vascular disease, bony tissue defects, recurrent Crohn’s fistulae, and chronic skin wounds has also been shown[13,14,15,16,17,18]. While these properties of ASCs hold promise for wide-ranging therapeutic applications, current methods used for isolation, expansion, and cryopreservation of ASCs typically employ undefined components such as serum and animal products, which render the resulting ASCs unsuitable for therapeutic purposes[19,20]. Although serum provides numerous factors that are important for cell attachment and growth, binding and neutralization of toxic molecules, reduction of shear stresses, and support of antioxidative defense, the undefined nature of sera makes downstream research and clinical applications of ASCs inconsistent and problematic. Furthermore, the use of serum is associated with risks of transmitting bacterial/viral infections and prions and inducing immunological reactions as a result of animal-borne components[21,22,23,24,25,26]. Consequently, the U.S. Food and Drug Administration (FDA) have issued strict guidelines against the use of xenoproducts[27]. Therefore, it is important to establish xeno-free defined protocols for different steps of ASC processing such as their isolation, expansion, and cryobanking. The objective of this study was to develop a simple but efficient xeno- and serum-free defined cryopreservation method for clinical-grade cryobanking of human ASCs by formulating and systematically testing defined cryopreservation media containing penetrating CPAs (i.e., dimethylsulfoxide [DMSO] and ethylene glycol [EG]), glass formers/polymers (i.e., trehalose, ficoll, and polyvinyl alcohol [PVA]), antioxidants (i.e., glutathione and ascorbic acid) and a calcium chelator (ethylene glycol tetraacetic acid [EGTA]) against the conventional undefined cryopreservation method. We chose to include antioxidants and polymers in order to mimic two beneficial aspects of serum supplementations (i.e., antioxidative defense and shear stress reduction) whereas combining two penetrating CPAs at lower concentration and chelating calcium can reduce CPA toxicity[28]. The post-thaw functionality and plasticity of cryopreserved ASCs were also examined.

## Materials and Methods

### Chemicals, Reagents, and Media

All reagents and media were purchased from Thermo Fisher Scientific (Waltham, MA, USA) unless otherwise stated. KnockOut Dulbecco’s modified Eagle’s medium (DMEM)/F-12 mixture containing 1X glutamax (2 mM L-alanine-L-glutamine), 10% fetal bovine serum (FBS), and 1% antibiotic/antimycotic mix served as culture medium. Unprocessed adipose tissue samples were transferred and held in Leibovitz’s L-15 medium containing 1% antibiotic/antimycotic mix until mechanically dissociated. Hank’s balanced salt solution (HBSS) including calcium, magnesium, and 1% antibiotic/antimycotic mix without phenol red was used for washing disaggregated adipose tissue samples. This HBSS solution was also used for preparation of collagenase type 1 (Worthington, Lakewood, NJ) solution. To inactivate collagenase, a 1,000X solution (0.1 M) of ethylenediaminetetraacetic acid (EDTA, Sigma, St. Louis, MO) was prepared in Dulbecco’s calcium- and magnesium-free phosphate-buffered saline (DPBS). To remove red blood cells, a red blood lysis buffer was prepared by dissolving 155 mM NH_4_Cl (Sigma), 10 mM KHCO_3_ (Sigma), and 1mM EDTA in ultrapure water (pH 7.3) and sterilized by filtering through a 2-μm polyethersulfone (PES) membrane filter. Ficoll-paque premium 1.073 (GE Healthcare, Atlanta, GA) was used for separation of mesenchymal stem cells by forming a density gradient. For magnetic cell sorting (MACS), FITC-conjugated monoclonal anti-CD31 and anti-CD45 antibodies, anti-FITC microbeads, column buffer (PBS containing 0.5% FBS and 2 mM EDTA), and MACS LD columns were purchased from Miltenyi Biotec (San Diego, CA, USA). Several components of cryopreservation media including DMSO, EG, trehalose, ficoll, PVA, EGTA, and glutathione were purchased from Sigma except L-ascorbic acid 2-phosphate magnesium salt that was purchased from Wako (Osaka, Japan).

The adipogenic differentiation medium was DMEM/F-12 containing 3% FBS, 0.25 mM 1-methyl-3-isobutylxanthine (IBMX, Cayman Chemical, Ann Arbor, Michigan), 5 μM rosiglitazone (AdipoGen, San Diego, CA), 1 μM dexamethasone, 66 μM biotin (Sigma), 34 μM D-pantothenate (Chem-Impex International, Wood Dale, IL), and 200 nM human insulin (Sigma). The osteogenic differentiation medium was DMEM/F-12 containing 10% FBS, 100 nM dexamethasone, 10 mM ß-glycerophosphate (Chem-Impex International), and 0.05 mM L-ascorbic acid-2-phosphate. For chondrogenic differentiation, DMEM/F-12 containing 1% FBS, 100 nM dexamethasone, 10 ng/mL TGF-ß1, 500 ng/mL BMP-6, 0.16 mM L-ascorbic acid-2-phosphate, 1% glutamax, 1% Insulin-Transferrin-Selenium-Ethanolamine (ITS-X), and 1% antibiotic/antimycotic mix was used. Chemicals for fixative and staining solutions included paraformaldehyde (Sigma), glacial acetic acid (Sigma), isopropanol, Alizarin Red (Chem-Impex International), Oil Red-O (Chem-Impex International), and Alcian Blue (Sigma).

### Isolation of ASCs

ASCs were isolated either from lipoaspirates or from non-disaggregated adipose tissue samples that were obtained from patients undergoing plastic surgery procedures at the Medical College of Georgia. The patient profile ranged between 27 and 76 years of age except for one case of a 13 year-old patient. The present study was approved by the Institutional Review Board (IRB) at Georgia Regents University (Protocol No. 668499–1). The IRB has determined that this study does not meet the definition of human subject research according to federal regulations 45CFR46 because all samples that were destined to be discarded otherwise were de-identified and acquired without any confidential information about patients. Therefore, no consent was needed as determined by the IRB.

To isolate ASCs, combinations of mechanical and enzymatic dissociation methods were used. The non-disaggregated adipose tissue samples were initially cut into small pieces under aseptic conditions and then further disaggregated using a gentleMACS dissociator (Miltenyi Biotec). The subsequent steps were identical for both types of adipose tissue samples (i.e., lipoaspirate and non-disaggregated adipose tissue). First, samples were washed with HBSS three to four times to remove excess blood cells. Next, the washed samples were digested with 0.1% collagenase solution in a shaking water bath at 37°C for 30 minutes. Upon inactivation of collagenase by adding 0.1 mM EDTA, the digested tissue was passed through a sterile strainer to remove undigested tissue pieces. To separate the stromal vascular fraction (SVF) from remaining fat/adipocytes, each filtered sample was centrifuged at 300 x *g* for 5 minutes, and the top layer of fat/adipocytes and the underlying layer of collagenase solution were then removed. The remaining pelleted SVF was resuspended in HBSS. After repeating the centrifugation step and discarding the supernatant, the SVF pellet was resuspended in red blood lysis buffer and incubated at room temperature for 10 minutes to eliminate red blood cells. Thereafter, the SVF was pelleted, resuspended in HBSS, and then passed through a 40-μm cell strainer. To preferentially isolate mesenchymal stem cells[29,30], the filtered SVF was then subjected to a density gradient separation using ficoll-paque premium according to the manufacturer’s instructions. Briefly, the SVF was layered onto ficoll-paque in a 15-ml or 50-ml tube at a 1.4:1 ratio and centrifuged at 400 x *g* for 30 minutes. The resulting interface containing primarily mesenchymal-type stem cells was recovered and washed in HBSS.

### Magnetic Sorting of ASCs

To further enrich mesenchymal stem cells, magnetic cell sorting was carried out using FITC-conjugated anti-CD31 and anti-CD45 antibodies, and MACS anti-FITC microbeads as suggested by the manufacturer. In brief, the cells gathered by density gradient separation were resuspended in column buffer and labeled with two above-mentioned FITC-conjugated antibodies at 4°C for 15 minutes. After removal of unbound antibodies, the cells were resuspended in fresh column buffer containing MACS anti-FITC microbeads and incubated at 4°C for 15 minutes. Subsequently, the cells were washed in the column buffer, resuspended in 500 μL of fresh column buffer, and applied to MACS LD columns placed onto a MidiMACS separation unit. The magnetically labeled CD31^+^ (endothelial) and CD45^+^ (leukocytes) cells were retained in the columns while unlabeled stem cells (CD31^-^CD45^-^ ASCs) passed through and were collected for subsequent use. The isolated ASCs were expanded through 4 to 9 passages before experimentation; however, cells from different passages or patients were not pooled for any experiments.

### Experimental Groups and Cryopreservation of ASCs

To develop a simple and efficient defined cryopreservation medium, we used HEPES-buffered DMEM/F-12 as a base medium and attempted to incorporate some of the protective effects of serum by adding two antioxidants (3 mM reduced glutathione and 5 mM ascorbic acid 2-phosphate) and two polymers (2% PVA and 5% ficoll) to this medium. The latter are expected to act as a macromolecule similar to serum albumin in terms of reducing shear stress and adhesion of cells to cryopreservation containers while providing some cryoprotective effects as well[31,32]. Further, 0.25 M trehalose was added to the cryopreservation medium based on its proven cryoprotective effect[33,34,35,36,37]. Other components added to the cryopreservation medium included two penetrating cryoprotective agents (CPAs) at two different concentrations (3.5% or 5% DMSO and EG) and a calcium chelator (0.1 mM EGTA). This approach seems to reduce CPA toxicity[28]. To facilitate experimental design, the concentration of trehalose, both polymers, and EGTA was fixed while the levels of penetrating CPAs and antioxidants in the cryopreservation medium were varied. In the first set of experiments, effects of two penetrating CPAs at a concentration of 3.5% and combinations of two antioxidants on the cryopreservation outcome were evaluated. The experimental groups included (1) conventional undefined freezing medium (i.e., 10% DMSO+10% DMEM/F12+80% FBS), (2) defined 1 freezing medium (i.e., 3.5% DMSO+3.5% EG in DMEM/F-12 containing 0.25 M trehalose, 2% PVA, 5% ficoll, and 0.1 mM EGTA), and defined 2 freezing medium (i.e., 3.5% DMSO+3.5% EG+3 mM reduced glutathione+5 mM ascorbic acid 2-phosphate in DMEM/F-12 containing 0.25 M trehalose, 2% PVA, 5% ficoll, and 0.1 mM EGTA). Untreated ASCs served as controls. Based on the outcome of the first set of experiments, the concentration of two penetrating CPAs was increased to 5% and compared to the lower concentration (3.5%) in a second set of experiments. Thus, the experimental groups were (1) undefined freezing medium (i.e., 10% DMSO+10% DMEM/F12+80% FBS), defined 2 freezing medium (i.e., 3.5% DMSO+3.5% EG+3 mM reduced glutathione+5 mM ascorbic acid 2-phosphate in DMEM/F-12 containing 0.25 M trehalose, 2% PVA, 5% ficoll, and 0.1 mM EGTA), and defined 3 freezing medium (i.e., 5% DMSO+5% EG+3 mM reduced glutathione+5 mM ascorbic acid 2-phosphate in DMEM/F-12 containing 0.25 M trehalose, 2% PVA, 5% ficoll, and 0.1 mM EGTA). The control group consisted of untreated ASCs. Two thawing rates (i.e., warming in air and in a water bath at 37°C) were tested for each experimental group whereas the cooling rate was not varied and was 1°C/min.

For cryopreservation, cells at passage number 4 to 9 were equilibrated with the cryopreservation media in one step at room temperature (RT) for 10 minutes. During the last 5 minutes of the equilibration time, ASCs were aspirated into ½-cc straws (TS Scientific, Perkasie, PA) and then both ends of the straws were heat-sealed. At the end of the equilibration time, the straws containing ASCs were transferred to a controlled-rate freezer (Planer KRYO 10 Series II, Planer PLC, Sunbury, United Kingdom) at 0°C and cooled to -7°C at 2°C/min. After seeding of extracellular ice and holding at -7°C for 10 min, the straws were cooled to -70°C at 1°C/min and then plunged into liquid nitrogen. For thawing, two different warming rates were tested by holding samples either in air or in a water bath at 37°C until ice disappeared. Upon thawing and removal of CPAs in three steps at RT by adding ASC medium for 5 minutes, the viability of cryopreserved ASCs was examined by trypan blue staining. Next, thawed ASCs were plated at a low density and their plating efficiency was determined after overnight culture according to the following formula: *% Plating efficiency = number of viable cells harvested/number of viable cells plated x 100*.

### Adipogenic Differentiation and Oil Red-O Staining

To induce adipogenic differentiation, ASCs were first grown to ~80% confluency, and the culture medium was then replaced with adipogenic differentiation medium (DMEM/F-12 containing 3% FBS, 0.25 mM IBMX, 5 μM rosiglitazone, 1 μM dexamethasone, 66 μM biotin, 34 μM D-pantothenate, and 200 nM human insulin). Upon 3 days of adipogenic induction, the cells were switched to adipocyte maintenance medium that was prepared similar to the adipogenic differentiation medium without adding IBMX and rosiglitazone. After 11 additional days of culture in the adipocyte maintenance medium, differentiation into adipogenic lineage was evaluated by examining lipid formation using Oil Red-O staining. To do so, the cells were fixed in 4% paraformaldehyde at RT for 30 minutes and then rinsed three times with distilled water. After an additional wash with 60% isopropanol, the cells were stained with 0.3% Oil Red-O solution at RT for 15 minutes. Subsequently, the stained cells were again rinsed three times with distilled water and examined using an inverted microscope.

### Osteogenic Differentiation and Alizarin Red Staining

At ~80% confluency, ASCs were induced to osteogenic differentiation by replacing the culture medium with osteogenic differentiation medium (DMEM/F-12 containing 10% FBS, 100 nM dexamethasone, 10 mM ß-glycerophosphate, and 0.05 mM L-ascorbic acid-2-phosphate). The cells were cultured in the osteogenic differentiation medium for 3 weeks while replacing the medium every 3 days. The cells were then fixed in 4% paraformaldehyde at RT for 30 minutes, and the mineralization was assessed by Alizarin Red staining. To stain the fixed cells, 2% Alizarin Red solution was first prepared in distilled water, and pH was adjusted to 4.3. Next, the fixed cells were incubated with the Alizarin Red solution at RT in the dark for 45 minutes. Thereafter, the stained cells were washed four times with distilled water and then covered with DPBS for microscopic examination.

### Chondrogenic Differentiation

To induce chondrogenic differentiation of ASCs, a pellet culture method was used along with chondrogenic induction medium (DMEM/F-12 containing 1% FBS, 100 nM dexamethasone, 10 ng/mL TGF-ß1, 500 ng/mL BMP-6, 0.16 mM L-ascorbic acid-2-phosphate, 1% glutamax, 1% Insulin-Transferrin-Selenium-Ethanolamine (ITS-X), and 1% antibiotic/antimycotic). Approximately 200,000 cells suspended in the induction medium were transferred to a 15-ml polypropylene tube and gently pelleted at 150 x *g* for 5 minutes. The pelleted cells were then cultured within the tube at 37°C under 5% CO_2_ in air for 3 weeks, with a medium change every 3 days. The cap of the tube was loosened during the culture period to allow air exchange. At the end of the culture period, the spheroid pellet in each tube was fixed in 4% paraformaldehyde at RT for 30 minutes and then washed two times with distilled water before incubating in 1% Alcian Blue staining solution at RT for 30 minutes. Next, the spheroids were washed with a destaining solution (6:4 mix of ethanol and acetic acid) and examined under a dissecting microscope.

### Karyotyping

The karyotyping protocol was described elsewhere in detail[38]. Briefly, to confirm chromosomal stability of ASCs cryopreserved using the defined 3 protocol, the thawed cells were plated and cultured for 4 days before incubating with colcemid (0.1 μg/mL) at 37°C overnight. Next, the cells were harvested, incubated in hypotonic KCl solution at 37°C for 20 min, and fixed by adding Carnoys fixative drop-wise to a final volume of 10 ml. The cells were held on ice for 10 min, and the fixation procedure was then repeated through two more cycles. The fixed cells were dropped on pre-cleaned slides and air dried. Giemsa stain was used for G-banding and at least 20 metaphase spreads were evaluated to determine the chromosomal normality of the cryopreserved cells.

### Statistical Analysis

Experiments in each series were repeated at least three times, and data reported are means of experimental repeats involving viability and plating efficiency rates with error bars representing standard error of mean (SEM). The data were analyzed by ANOVA/Tukey’s pairwise comparison test using GraphPad Prism (GraphPad Software, Inc., San Diego, CA). Before ANOVA, arcsine transformation was performed on proportional data. Differences between the groups were considered statistically significant when the *p*-value was less than 0.05.

## Results

### Antioxidant Supplementation Improves the Cryopreservation Outcome

**Fig 1:**
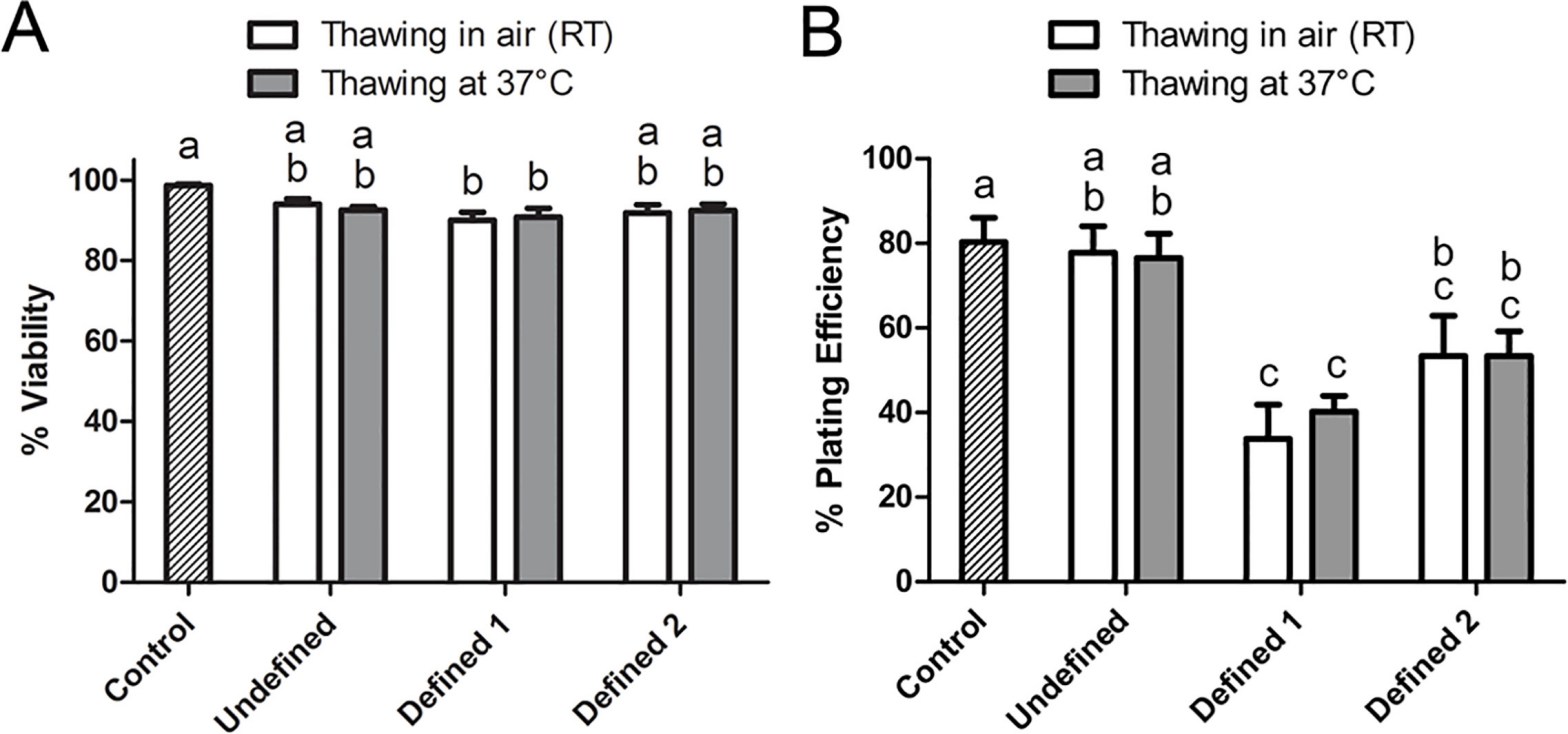
The post-thaw viability (A) and plating efficiency (B) of hASCs cryopreserved using undefined and defined cryopreservation media. The conventional undefined cryopreservation medium consisted of 10% DMSO+10% DMEM/F12+80% FBS while defined 1 freezing medium was composed of 3.5% DMSO+3.5% EG in DMEM/F-12 containing 0.25 M trehalose, 2% PVA, 5% ficoll, and 0.1 mM EGTA. Defined 2 freezing medium was similar to defined 1 medium but additionally contained two antioxidants (i.e., 3 mM reduced glutathione+5 mM ascorbic acid 2-phosphate). Data shown are mean±SEM. Columns with different letters in each thawing category are significantly different (p < 0.05).

In this set of experiments, the post-thaw viability and plating efficiency of ASCs cryopreserved under defined conditions were evaluated with respect to those of the conventional undefined method as explained in subsection 2.4. The results are summarized in Fig 1. The post-thaw viability was high in all groups and varied between 90.0% and 98.7% (Fig 1A). Two different thawing rates (warming in air and in a water bath at 37°C) did not significantly change the post-thaw viability. However, the combination of EG and DMSO at 3.5% concentration (3.5%) in the absence of antioxidants (Defined 1) yielded significantly lower viability rates after thawing in air and at 37°C (90.0% and 90.8%, respectively) compared to untreated controls (98.7%) while these viability rates were comparable to those of 10% DMSO in the undefined medium (94.0% and 92.5%, respectively). Addition of two antioxidants (reduced glutathione and ascorbic acid 2-phosphate) to the Defined 1 freezing medium (now termed as Defined 2) improved the viability to the level of controls after thawing both in air (91.8%) and at 37°C (92.4%). Unlike the post-thaw viability, the plating efficiency rates after thawing in air and at 37°C were significantly lower in the Defined 1 group (33.8% and 40.2%, respectively) compared to those (77.8% and 76.5%, respectively) of the undefined medium whereas addition of antioxidants (Defined 2) improved the plating efficiency rates (53.4% and 53.4%, respectively), such that there was no significant difference between the Defined 2 and undefined group. However, the plating efficiency rates in the Defined 2 group were still significantly lower than that (80.3%) of the control group.

### Defined Cryopreservation Method Yields Viability and Plating Efficiency Rates Comparable to the Conventional Undefined Method

**Fig 2:**
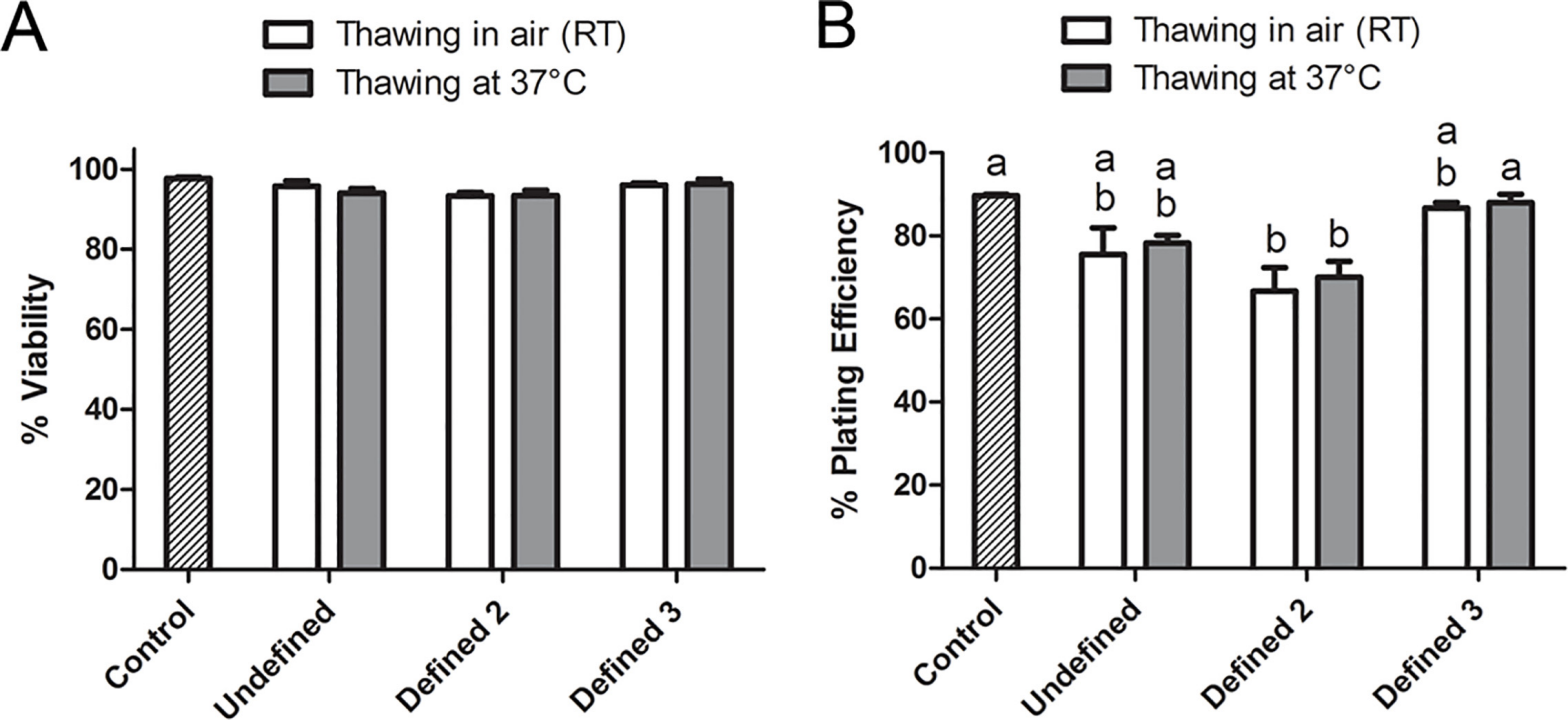
The post-thaw viability (A) and plating efficiency (B) of hASCs after further optimization of the defined cryopreservation medium. The undefined cryopreservation medium consisted of 10% DMSO+10% DMEM/F12+80% FBS while defined 2 medium was made up of 3.5% DMSO+3.5% EG+3 mM reduced glutathione+5 mM ascorbic acid 2-phosphate in DMEM/F-12 containing 0.25 M trehalose, 2% PVA, 5% ficoll, and 0.1 mM EGTA. Defined 3 cryopreservation medium had a slightly increased concentration (5%) of DMSO and EG; otherwise it was similar to defined 2 medium). Data shown are mean±SEM. Columns with different letters in each thawing category are significantly different (p < 0.05).

Based on the results of the first set of experiments, the Defined 2 cryopreservation medium was modified in this set of experiments by increasing concentrations of two penetrating CPAs (EG and DMSO) from 3.5% to 5% (now referred to as Defined 3). Hence, the Undefined, Defined 2, and Defined 3 cryopreservation media were tested against each other as well as against untreated controls. The results of these experiments are summarized in Fig 2. The post-thaw viability was high in all groups and varied between 93.3% and 97.7% without any significant difference between the control and experimental groups (Fig 2A). In contrast, the plating efficiency rates after thawing in air and at 37°C were, respectively, 66.7% and 70.0% for the Defined 2 medium and significantly lower than that (89.7%) of untreated controls (Fig 2B). Nevertheless, these rates were comparable to those of the Undefined medium (75.5% and 78.3%, respectively). Notably, increasing the concentration of EG and DMSO to 5% (Defined 3) further improved the plating efficiency rates (86.7% and 88.0% after thawing in air and at 37°C, respectively) to the level of the control group.

### ASCs Cryopreserved Under Defined Conditions Maintain Their Plasticity

**Fig 3:**
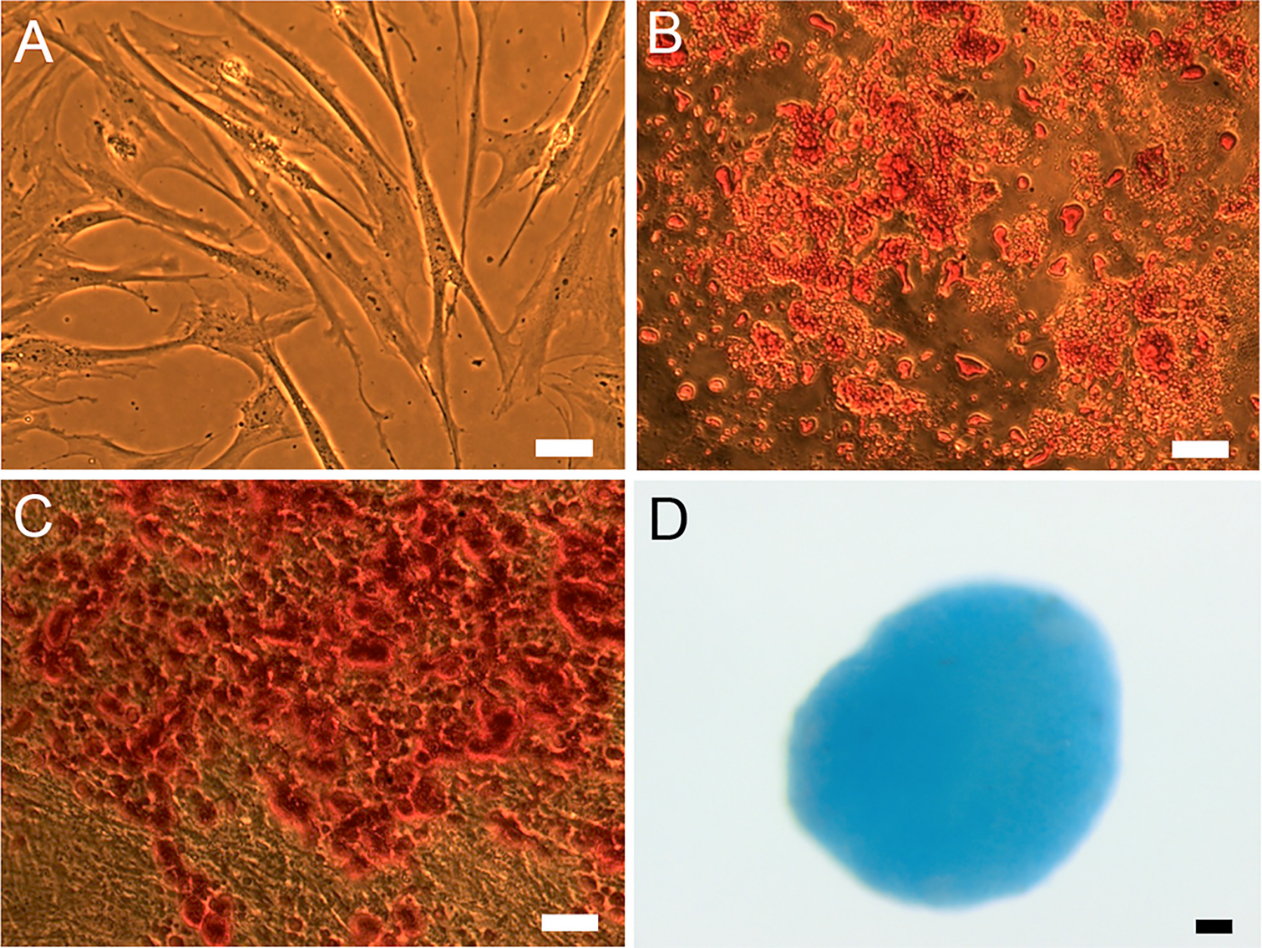
Representative images of ASCs in culture prior to differentiation (A) and after both cryopreservation and differentiation into adipogenic (B), osteogenic (C), and chondrogenic (D) lineages as shown by Oil Red-O, Alizarin Red, and Alcian Blue staining, respectively. In this set of experiments, ASCs were cryopreserved using defined 3 medium (5% DMSO+5% EG+3 mM reduced glutathione+5 mM ascorbic acid 2-phosphate in DMEM/F-12 containing 0.25 M trehalose, 2% PVA, 5% ficoll, and 0.1 mM EGTA). Scale bar = 50 μm.

To examine whether multipotency can be retained after serum-free defined cryopreservation, ASCs were subjected to freezing and thawing in the Defined 3 medium, induced to differentiate into adipogenic, osteogenic, and chondrogenic lineage along with unfrozen controls and subsequently stained with Oil Red-O, Alizarin Red, and Alcian Blue to detect lipid accumulation, extracellular mineralization, and proteoglycans, respectively. The specificity of all three staining protocols was verified by subjecting undifferentiated cells to each staining protocol. Microscopic examination of the stained samples revealed that upon inducing differentiation into adipogenic, osteogenic, and chondrogenic lineage by the respective induction media, both unfrozen controls and cryopreserved ASCs extensively differentiated into all three lineages as indicated by the lineage specific staining (Fig 3). These results suggest that cryopreserved ASCs maintain their plasticity.

### ASCs Cryopreserved Under Defined Conditions Retain Their Chromosomal Normality

**Fig 4:**
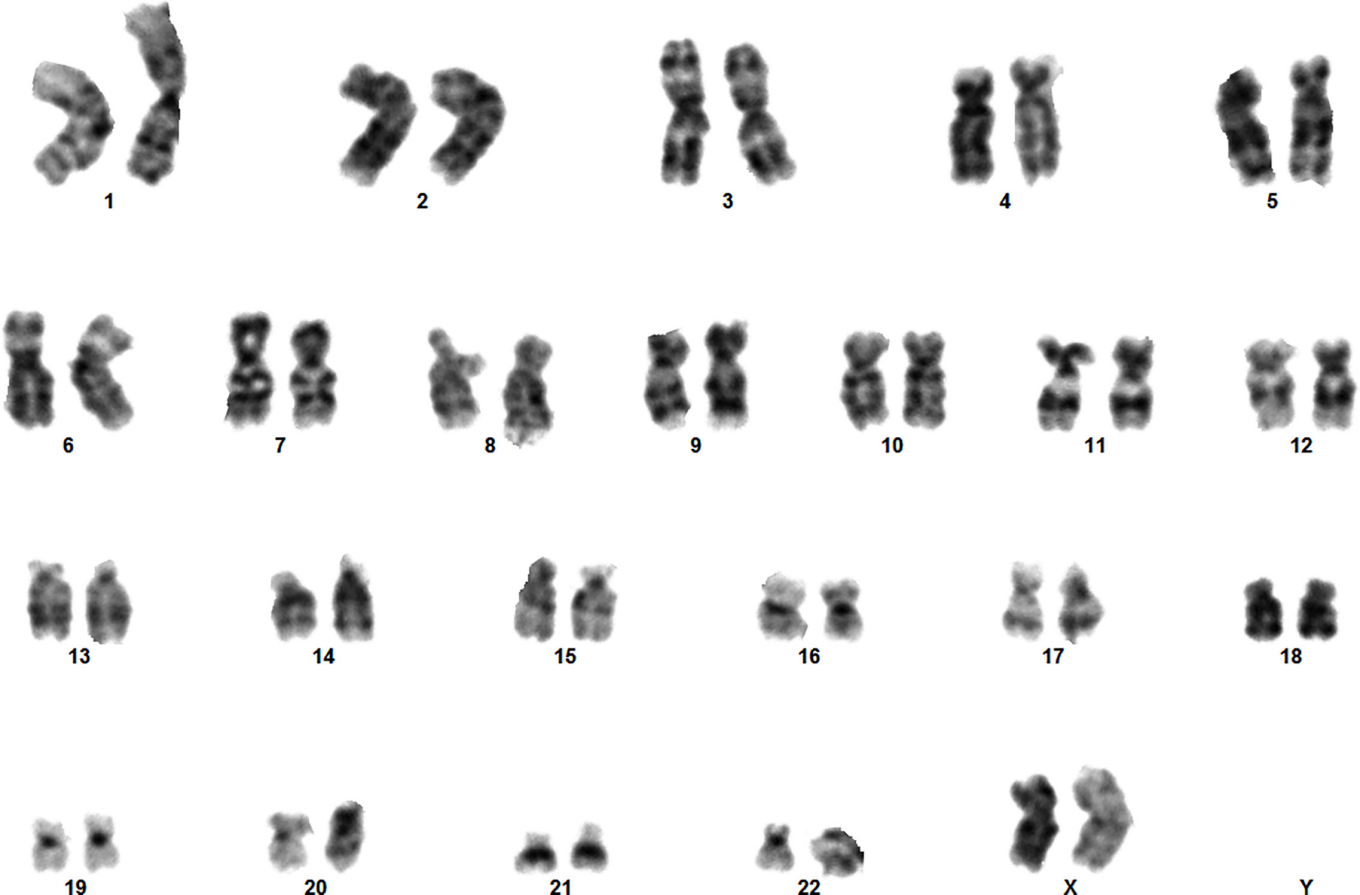
Normal human female karyotype of ASCs cryopreserved under defined conditions at passage 6 using defined 3 cryopreservation medium (5% DMSO+5% EG+3 mM reduced glutathione+5 mM ascorbic acid 2-phosphate in DMEM/F-12 containing 0.25 M trehalose, 2% PVA, 5% ficoll, and 0.1 mM EGTA).

To test whether the serum-free defined cryopreservation method induces any chromosomal abnormalities, ASCs underwent two freeze-thaw cycles at different passage numbers and were then karyotyped as explained in the subsection 2.8. As shown in Fig 4, ASCs were karyotypically normal after cryopreservation in the serum-free Defined 3 medium.

## Discussion

This study presents a serum- and xeno-free defined cryopreservation method for human ASCs by showing that ASCs maintain their viability, functionality, and plasticity similar to controls when cryopreserved in HEPES-buffered DMEM/F-12 medium containing a 5%-concentration of two permeating CPAs (EG and DMSO), a disaccharide (trehalose), two polymers (PVA and ficoll), a calcium chelator (EGTA), and two antioxidants (reduced glutathione and ascorbic acid 2-phosphate). Furthermore, the defined cryopreservation method supports retention of a normal karyotype. The results of the present study suggest that human ASCs can be cryobanked under defined conditions with success rates similar to those achievable by the conventional undefined cryopreservation. This is of significance for realization of diverse clinical applications of ASCs.

Studies on cryopreservation of ASCs are relatively few. Typically, ASCs are cryopreserved using 10% DMSO prepared mainly in pooled animal serum [20,39,40] that provides several beneficial nutrients and molecules supporting cell functionality, membrane integrity, antioxidative defense, and shear stress reduction while boosting buffer capacity. Indeed, the undefined medium in the present study, which contained 10% DMSO and 80% FBS, afforded good post-thaw viability and plating efficiency rates. Although such an undefined cryopreservation medium might be acceptable for some research purposes, clinical-grade cryobanking of stem cells preferentially requires defined xeno-free cryopreservation medium to avoid risks of transmitting infectious diseases, prions, or exposure to antigens that may induce immunological reactions[21,22,23,24,25,26]. To this end, we attempted to partially mimic antioxidative and shear stress-reducing aspects of serum by including two antioxidants and two polymers in our cryopreservation medium. Cryopreservation-associated events such as cooling and exposure to chemically toxic CPAs are expected to result in disrupted membrane transport and ionic imbalance leading to increased free radical formation and thus to lipid peroxidation, protein oxidation, DNA damage, and ultimately cell death. In fact, several studies reported increased free radical formation after cryopreservation[41,42,43,44] while addition of antioxidants to cryopreservation media proved to be beneficial to varying extents[41,45,46,47,48]. Consistent with these findings, supplementation of our defined medium with two antioxidants improved the cryopreservation outcome even though some components of the base medium might have acted as antioxidants (e.g., methionine, tryptophan, cysteine, tyrosine, ascorbic acid [49]) and thus partially masked the beneficial effect. Since the objective of the present study was to develop a defined cryopreservation method with a streamlined experimental design, individual and synergistic effects of different antioxidant were not systematically examined. Nevertheless, our findings warrant further detailed studies on optimization of an antioxidant cocktail that may be even more beneficial for serum-free defined cryopreservation of clinically relevant cell types.

In the present study, we have not systematically tested the beneficial effect of polymers. The rationale to include two polymers was to mimic shear stress-reducing and anti-adhesive effects of sera. Indeed, PVA has been employed with some success as a substitute for serum in embryo culture and cryopreservation media whereas ficoll was successfully used as a glass former in some vitrification media[32,50,51]. In addition, PVA was found effective in blocking heterogeneous ice nucleation[52]. However, it should be noted that other polymers may have similar protective effects. In fact, a recent study reported that 10% polyvinylpyrrolidone (PVP) alone afforded good post-thaw survival rates in a serum-free medium, albeit significantly lower than that obtained using 10% DMSO plus 80% serum[53]. Therefore, it might be worthwhile to systematically compare the beneficial effect of different polymers in order to further optimize defined cryopreservation media in future studies.

Cooling and warming rates are considered among the key factors determining cryosurvival. In case of slow freezing protocols, both fast and slow cooling rates can be detrimental as a result of intracellular ice formation (IIF) and solution effects, respectively as explained by the two-factor hypothesis[54,55]. Permeating CPAs such as DMSO protect cells against both modes of cryoinjury by suppressing IIF and reducing electrolyte concentration in the unfrozen fraction of cryopreservation medium[56]. The cooling rate used in the present study (i.e., 1°C/min) is achievable without a controlled-rate freezer and produced satisfactory results. Therefore, no further optimization has been attempted. Instead, combinations of two permeating CPAs (DMSO and EG) and two thawing rates were tested. The best results, comparable to unfrozen controls, were obtained when 5% concentrations of both CPAs were used. Among the permeating CPAs, EG is regarded to be less toxic to mammalian cells[57,58,59]. Therefore, combinations of 5% concentrations of DMSO and EG can be considered as a better alternative to 10% DMSO alone[28]. Interestingly, two thawing rates yielded similar results. Nonetheless, the faster thawing rate might be preferable to avoid extended exposure to CPAs and non-physiologic conditions when working with large volumes of cells. It is also worth noting that based on trypan blue exclusion assay, decent post-thaw viability rates were obtained using lower concentrations (3.5%) of both penetrating CPAs, even in the absence of antioxidants (Fig 1A). However, further examination of the membrane-intact cells by a plating efficiency assay revealed that a significant portion of the trypan-blue-impermeable cells were nonfunctional (Fig 1B). These findings indicate that membrane integrity assays are helpful but not sufficient to assess the true viability of cryopreserved cells. To uncover sublethal injuries affecting long-term viability and functionality of cryopreserved cells, additional evaluation criteria/assays should be implemented. This is particularly critical for clinical application of cryobanked stem cells. In the present study, the viability and functionality of cryopreserved ASCs were evaluated side-by-side using both trypan blue exclusion and plating efficiency assays. The latter is known as a sensitive assay and can assess both viability and functionality of cryopreserved cells. Furthermore, the plasticity of cryopreserved ASCs was confirmed in the present study by inducing their differentiation into adipogenic, chondrogenic, and osteogenic lineages. However, the differentiation potential of ASCs was confirmed by microscopic observations, not by quantification of the differentiated cells due to associated challenges. Nevertheless, combination of all three approaches is expected to yield an accurate picture of the cryopreservation outcome.

The suppression of IIF can also be achieved by adding non-reducing sugars such as trehalose to cryopreservation media. Trehalose is an excellent glass former[60,61,62] and acts as an osmolyte/protectant against osmotic, chemical, oxidative and hypoxic stresses [63,64,65,66,67,68]. Although trehalose affords its maximum protection when present both intra- and extracellularly[33,34,61], combination of extracellular trehalose with a penetrating CPA also proved to be efficient[35,37,69]. Based on its proven protective effect, trehalose was also included in the defined medium; however, a systematic evaluation of trehalose concentration might be helpful to further optimize and reduce concentrations of the two permeating CPAs as well.

In conclusion, cryobanking of ASCs under defined conditions is achievable with success rates similar to those obtained using undefined cryopreservation media. Considering advances in tissue engineering and regenerative medicine, development of a successful serum- and xeno-free defined cryopreservation method represents a significant progress towards therapeutic applications of stem cells.
